# Digital learning designs in physiotherapy education: a systematic review and meta-analysis

**DOI:** 10.1186/s12909-020-02483-w

**Published:** 2021-01-13

**Authors:** Nina Bjerketveit Ødegaard, Hilde Tinderholt Myrhaug, Tone Dahl-Michelsen, Yngve Røe

**Affiliations:** 1 Department of Physiotherapy, Oslo Metropolitan University, Post Box 4. St. Olavsplass, 0130 Oslo, Norway; 2 Department of Nursing and Health Promotion, Oslo Metropolitan University, Post Box 4. St. Olavsplass, 0130 Oslo, Norway

**Keywords:** Digital learning designs, Digital learning technology, Physiotherapy education, Learning outcomes, Systematic review, Meta-analysis

## Abstract

**Background:**

Digital learning designs have the potential to support teaching and learning within higher education. However, the research on digital learning designs within physiotherapy education is limited. This study aims to identify and investigate the effectiveness of digital learning designs in physiotherapy education.

**Methods:**

The study was designed as a systematic review and meta-analysis of randomized and non-randomized trials. A search of eight databases on digital learning designs and technology was conducted. Study selection, methodology and quality assessment were performed independently by three reviewers. The included studies were mapped according to the types of digital interventions and studies. For similar interventions, the learning effects were calculated using meta-analyses.

**Results:**

Altogether, 22 studies were included in the review (17 randomized controlled trials and five cohort studies). A blended learning design was used in 21 studies, a flipped classroom model in five and a distance learning design in one. Altogether, 10 of the 22 articles were included in meta-analyses, which showed statistically significant effects for flipped classrooms on knowledge acquisition (standardized mean difference [SMD]: 0.41; 95% confidence interval [CI]: 0.20, 0.62), for interactive websites or applications (apps) on practical skills (SMD: 1.07; 95% CI: 0.71,1.43) and for students self-produced videos on a practical skill in a cervical spine scenario (SMD: 0.49; 95% CI: 0.06, 0.93). Overall, the effects indicated that blended learning designs are equally as or more effective than traditional classroom teaching to achieve learning outcomes. Distance learning showed no significant differences compared to traditional classroom teaching.

**Conclusions:**

The current findings from physiotherapy education indicate that digital learning designs in the form of blended learning and distance learning were equally or more effective compared to traditional teaching. The meta-analyses revealed significant effects on student learning in favour of the interventions using flipped classrooms, interactive websites/apps and students self-produced videos. However, these results must be confirmed in larger controlled trials. Further, research should investigate how digital learning designs can facilitate students’ learning of practical skills and behaviour, learning retention and approaches to studying as well as references for teaching and learning in digital learning environments.

**Supplementary Information:**

The online version contains supplementary material available at 10.1186/s12909-020-02483-w.

## Background

During the past decade, digital learning designs have been increasingly used in teaching practices in higher education. UNESCO [1] emphasizes that digital learning can transform teaching practises, improve the quality and enhance the sustainability of higher education. A digital learning design has been described as a didactic plan that integrates digital learning technology to support students’ learning processes and to achieve constructive alignment between learning outcomes, teaching and learning activities and feedback and assessment methods [2]. The designs can fully or partly integrate digital learning tools and resources (e.g., video lectures or video tutorials) and have the potential to move traditional teaching out of the classroom and to facilitate active learning in the classroom [3]. The various designs provide opportunities to improve self-regulating abilities, facilitate active learning and make the learning process more transparent [4].

Digital learning designs encompass various technologies such as virtual reality, podcasts, apps, serious/educational games, 360° video and animations. These technologies can be directly implemented in the learning activities or combined with other planned learning activities. Because no conceptual framework for digital learning designs exists, similar digital learning designs are often mentioned using different terminology. Digital learning designs can be divided into blended learning (e.g., flipped classrooms) and distance learning (e.g., fully e-learning courses). The main difference is that blended learning combines online and face-to-face teaching and often combine both synchronous learning (real-time, in-person or online) and asynchronous learning (flexible time, online), whereas distance learning is used as a synonym for fully online learning. In distance learning, teaching and learning is facilitated by a web-based system to connect learners, resources and teachers; and it can be completely asynchronous (flexible regarding when the student is online) [5].

There is conflicting evidence of the effectiveness of the different digital learning designs used in physiotherapy and other health professions education. A systematic review on the effects of the flipped classroom approach for the education of health profession students did not reveal compelling evidence for the effectiveness of the method for improving academic outcomes compared to traditional teaching [6]. In contrast, a meta-analysis on the effectiveness of flipped classrooms in health professions education concluded that this approach yields a significant improvement in student learning compared with traditional teaching methods [7]. Another systematic review on blended learning in health professions showed that it has the potential to improve clinical competence among health students and to be more effective than or at least as effective as non-blended learning for knowledge acquisition [8]. In contrast, a systematic review and meta-analysis of the effectiveness of computer-assisted instruction (CAI) to teach physical examination in health science education found no consistent benefit of using this method [9].

Graduation from a physiotherapy programme qualifies the graduate for practice as an independent and autonomous professional [10]. The physiotherapy curriculum is characterized by a combination of theory, skills training and practice [11]. Until now, digital learning designs in physiotherapy education have been criticised for not being grounded in a theoretical learning perspective [12]. A systematic review on online technology use (e.g., websites and discussion boards) in physiotherapy education concluded that these technologies enhanced practical skills performance, knowledge acquisition and the development of critical and reflective thinking [13]. Another systematic review on the role of computer-assisted learning in physiotherapy education, concluded that it was largely under-researched compared to other health professions education [14]. To our knowledge, no recent review on digital learning designs in physiotherapy education have been conducted. The aim of this systematic review is to identify and investigate the effectiveness of various digital learning designs in physiotherapy education.

## Methods

This systematic review was carried out according to the Preferred Reporting Items for Systematic reviews and Meta-Analysis guidelines [15]. The protocol of the systematic review was registered in the international prospective register of systematic reviews (PROSPERO; https://www.crd.york.ac.uk/prospero) with registration number CRD42019134917.

We included randomized controlled trials (RCTs) and cohort studies that reported baseline and post-treatment measures and for both study groups and that met the following criteria: (a) a study population of physiotherapy students in a physiotherapy education programme (bachelor’s/undergraduate, masters/ entry level, Doctor of Physical Therapy [DPT] or Doctor of Philosophy [PhD]); (b) assessed the learning outcomes of a digital learning design (e.g., flipped classroom); (c) compared the outcomes to traditional classroom teaching; and (d) reported on students’ final grades and self-reported learning outcomes (e.g., students’ perceptions, motivation, attendance, commitment, engagement and satisfaction with the learning design). We included only studies with summative assessments for the final exam to measure knowledge, skills or affective learning outcomes (e.g., values, attitudes and behaviours) [16]. The exclusion criteria were studies where less than half of the study population were physiotherapy students, that were aimed to train graduated physiotherapists for work life (e.g., courses and seminars that did not provide credits), where the use of digital learning technology was not part of an explicit learning strategy and in languages other than English or Scandinavian.

### Search strategy

Two information specialists (MWG, EK) searched Medline, Cinahl, Education Resources Information Center, Education Source, Scopus, Teacher Reference Center, Embase and Cochrane Central. The publication period was limited to 1 January 2010 to 28 August 2020. Because there are limited uses of learning designs in physiotherapy education before 2010, we chose to limit the search to articles published since 2010. Examples of search terms were ‘assisted instruction/education’, ‘distance educational, technology/webcasts/information, technology/multimedia/computer, user training/world wide web, applications/computer simulation’, ‘blended’, ‘e-learning’, ‘m-learning’, ‘web-based’, ‘virtual’, ‘streaming’, ‘interactive’, ‘hybrid’, ‘gaming’, ‘massive open online course’, ‘flipped’ and ‘simulation’. The complete search strategy is shown in Additional File 1.

### Selection of articles and data extraction

Three reviewers (N.BØ, H.TM, Y.R) independently screened the titles and abstracts from the literature search according to the selection criteria using the Rayyan website/app as a screening tool [17]. The full text of the relevant articles was assessed independently by these reviewers. The full-text articles that met the inclusion criteria were included in the review. Disagreement on selection of articles was solved by discussion until a consensus was reached.

The following data were extracted from the included studies by the first author (N.BØ) and cross-checked by the other two reviewers (H.TM and Y.R): authors of the study, publication year, country, study design, characteristics of the population (e.g., level of education), characteristics of the interventions (blended or distance learning designs), comparison to traditional classroom teaching and outcomes (e.g., grades and method of assessment). The final decision on the articles included was made via a discussion meeting attended by all authors.

### Risk of bias assessment

We assessed the risk of bias for the included RCTs and cohort studies using Cochrane’s risk of bias tool [18]. The risk of bias assessment was conducted by three reviewers (N.BØ, H.TM, Y.R) independently. Bias was assessed as high, low or unclear for the five domains: selection, performance, attrition, reporting and other potential threats to validity [18].

### Data analysis

Due to the multiple terms used for digital learning designs, an overview of some of the most used terms are included in Table 1.

**Table 1:** Overview of commonly used digital learning design concepts

Blended learning	Distance learning
Blended learning “is the thoughtful integration of classroom face-to-face learning experiences with online learning experiences. There is considerable intuitive appeal to the concept of integrating the strengths of synchronous (face-to-face) and asynchronous (flexible-time) learning activities [19] (p. 96).	Distance learning is a “planned learning that normally occurs in a different place from teaching and as a result requires special techniques of course design, special instructional techniques, special methods of communication by electronic and other technology, as well as special organizational and administrative arrangements [20] (p. 2).
**Flipped classroom model** In a flipped classroom, “the information transmission component of a traditional face-to-face lecture (‘traditional lecture’) is moved out of class and the learning in-class are active, collaborative tasks. Students prepare for class by engaging with resources that cover what would have been in a traditional lecture. After class they follow up and consolidate their knowledge” [21] (p. 1)	**E-learning courses** a structured course delivered electronically with different elements: live or pre-recorded lecture content, video, quizzes, simulations, games, activities, and other interactive elements. E-learning can also be facilitated as virtual classrooms - a type of online learning in which live interaction between instructors and participants take place synchronous.
**Blended learning on and off campus** An example of blended learning design is where the students, for example, gain access to digital learning resources prior to in-class teaching and/or after classroom teaching, but the teaching is traditionally offered. Another example is that the learning activities in the classroom teaching are given and answered through digital learning technology and software.	**Mobile learning (m-learning**) Variant of e-learning; teaching takes place via mobile equipment, e.g. mobile smart phones. M-learning is “the processes of coming to know through conversations across multiple contexts among people and personal interactive technologies” [22] (p. 225)
**Hybrid Learning** Educational model where one student group follows the course on campus and simultaneously individuals follow the course remotely through digital technology. Hybrid learning can combine synchronous learning with asynchronous learning elements like e.g. online forums, discussion boards. Hybrid classrooms vary widely according to the subject matter taught and the needs of specific groups of learners.	**Remote/at-home learning** A course designed to be delivered online, not intended to meet in-person, students intended not to work on assignments in the same space, and do not attend lectures or classes virtually with video or audio communication to participate.
	**Massive open online courses (MOOC) “**A massive open online course is an online course aimed at unlimited participation and open access via the web” [23], (p. 442). “MOOC integrates the connectivity of social networking, the facilitation of an acknowledged expert in a field of study, and a collection of freely accessible online resources. The learners are typically adults and self-organize their participation according to learning goals, prior knowledge and skills, and common interests” [24] (p. 4)

First, the included articles were categorized according to the study design. Thereafter, the descriptions of the learning designs, the digital learning technologies used, and the learning outcomes were considered to pool the results in the meta-analyses based on their similarities. We calculated mean differences for pooling similar continuous outcomes (e.g., students’ satisfaction with the learning design reported on a Likert scale of 1–5), and we used standardized mean differences (SMDs) when the included studies used different scales for the same outcome. For all outcomes, we reported the associated 95% confidence intervals (CIs). Double-data entries were performed. The meta-analysis was based on a random effects model, as we expected heterogeneity across the included studies. Studies that reported similar populations, interventions and outcomes were pooled in the meta-analyses. For studies that were too heterogeneous for pooling, we present the results narratively.

## Results

**Fig. 1:**
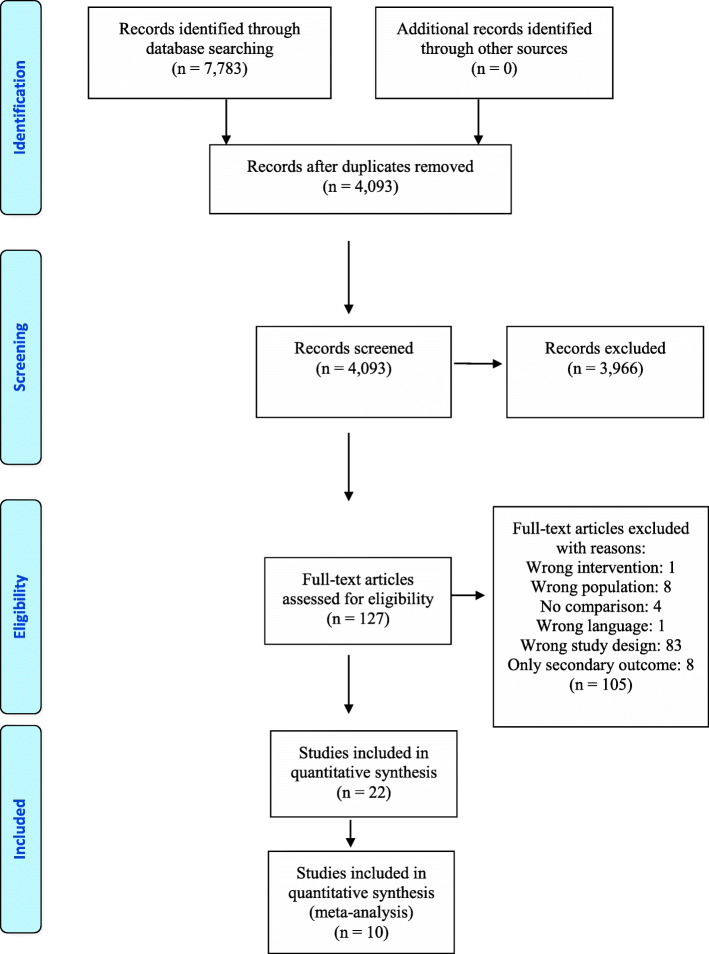
PRISMA flow chart of the records and study selection process

Altogether, we included 22 studies (Fig. 1) with a total of 2186 participants (study range: *n* = 16–176). The studies included students at the bachelor’s/undergraduates (*n* = 17), master’s/ entry level (*n* = 1) and DPT programme level (*n* = 4). Of the included studies, five were from Australia [25–29], five from Spain [30–34], three from Brazil [35–37], one from Denmark [38] and eight from the USA [39–46]. Seventeen of the studies had a RCT design [25, 27–42], and five were cohorts [26, 43–46].

All the studies were published between 2010 and 2020. A detailed overview of the included studies is shown in Table 2. For the 10 studies that were similar in terms of design, population, interventions and outcomes, we conducted meta-analyses using RevMan 5.3 software (Cochrane Community worldwide) [27, 28, 30–34, 44–46]. Twelve studies were too heterogeneous and were not included in the meta-analyses [25, 26, 29, 35–43]. They are described and summarized narratively in the text and Table 2.

**Table 2:** Characteristics of the included studies: randomized controlled trials (RCTs) and cohort studies

Author, year, country, study design	Population	Digital learning design, intervention, comparison	Outcome
Arroyo-Morales et al., 2012, [30] Spain, RCT	Under-graduate Physiotherapy students second year *n* = 46	**Digital learning design:** Blended learning **Context**: Theoretical acquisition and skills training on campus **Subject/skills:** Palpation and ultrasound examination of the knee joint **Duration:** In-class: two 2-h sessions, traditional lectures; self-studies: 20 h **Intervention:** In-class: traditional lectures; post-class: free access to the Ecofisio interactive website/app **Comparison:** In-class: two 2-h sessions, traditional lectures, access to documents and books on the topic **Both groups:** 3-week self-study period	**MCQ:** 20 MCQs (max 10 points); and assessed knowledge of ultrasound physics (5 questions), ultrasound technology (5questions), clinical applications (5 questions), and anatomy (5questions) **OSCE:** Skills in palpation and ultrasound imaging of the knee; grading system: 3 = excellent, 0 = incorrect (max 15 points each) Also measured the time taken by the student to generate a reliable ultrasound image and to localize a specific knee structure by palpation **Students’ evaluation:** Quality of the educational intervention: competence of the teacher, students’ acquisition of knowledge/skills, students’ interest in participating in the study for another anatomic region and—for the experimental group—satisfaction with the Ecofisio website; also asked whether they would have preferred to be in another study group; 5-point Likert scale (5 = strongly agree, 1 = disagree)
Bartlett and Smith, 2020, [39] USA, RCT	Under-graduate Physiotherapy students first year *n* = 20	**Digital learning design:** Blended learning **Context:** Skills training on campus **Subject/skills:** Cardiovascular and pulmonary physical therapy **Duration:** 45-min laboratory session **Intervention:** Mobile app only group and demonstration plus mobile app group. Mobile app only group: 5-min. Tutorial on how to navigate, no professorled demonstration of instruction of the clinical skills, then practiced the skills in a lab. Sessions. Demonstration plus mobile app group given the same demonstration and verbal information as the control group, take notes and ask questions. 5-min. Tutorial on how to navigate through the iPad and then participated in a lab. Sessions **Comparison:** Demonstration-only group: demonstration and practice of the skills in a laboratory session	**Practical exam:** Students tested on their psychomotor skills related to their ability to perform and interpret clinical skills; assessed using a mock patient not related to the study; 1 = satisfactory or 0 = unsatisfactory (max score 18); 3 examiners
Blackstock et al., 2013, [25] Australia, 2 RCTs	Under-graduate Physiotherapy students first year *n* = 349	**Digital learning design:** Blended learning **Context:** Simulation training on campus and clinical placement **Subject/skills:** Cardiorespiratory **Duration:** 4 weeks **Intervention 1:** Simulated learning environment videos; 1 week in the simulated learning environment, then 3 weeks in clinical immersion **Intervention 2:** 50% of day in the simulated learning environment and 50% in clinical immersion during the first 2 weeks (equal to 1 full-time simulation week), then 2 weeks in clinical immersion **Comparison:** 4 weeks in clinical immersion	**Practical exam:** Assessment of competency to practice in the cardio-respiratory field, measured using two clinical examinations based on the Assessment of Physiotherapy Practice; 7 key standards; score range: 0 = infrequently/rarely demonstrates performance indicators, 4 = demonstrates most performance indicators to an excellent standard, N/A = not applicable and not assessed **Students’ evaluation:** Scales for analysis of student’s self-rating of confidence with patients in communication, assessment and management; 13 Likert items; checked for reliability (Cronbach’s α)
Cantarero-Villanueva et al., 2012, [31] Spain, Single-blinded RCT	Under-graduate Physical therapy students, *n* = 44	**Digital learning design:** Blended learning **Context:** Theoretical acquisition and practical training on campus **Subject/skills:** Musculoskeletal palpation and ultrasound assessment of the lumbopelvic area **Duration:** 1 semester **Intervention:** 6 classroom hours (traditional lectures and practical training) and 20 self-study hours plus free access to an interactive website/app (Ecofisio) on musculoskeletal palpation and ultrasound assessment **Comparisons:** In-class: traditional lectures and practical training; 20 self-study hours: access to documents and books on the topic	**OSCE:** Ultrasound imaging, two components: musculoskeletal and skills in ultrasound imaging; grading system: 3 = excellent, 0 = incorrect; maximum score: 9 (musculoskeletal) and 15 (ultrasound imaging); validated **After OSCE:** Students invited to establish 2 additional measurements in the same model; graded one at a time using the same human model **Students’ evaluation:** Quality of the educational programme, 5-point Likert scale (5 = strongly agree, 1 = disagree); participant assessments included teacher’s competence, participants’ own acquisition of knowledge/skills, complexity of the knowledge/skills, possibility of participation using e-learning and (for the experimental group) satisfaction with the Ecofisio website
da Costa Vieira et al., 2017, [35] Brazil, Prospective crossover RCT	Under-graduate Physiotherapy students second to fourth year, *n* = 72	**Digital learning design:** Blended learning **Context:** Theoretical acquisition on campus **Subject/skills:** Physiotherapy in oncology **Duration:** 2 days and 6 modules (3 modules/day) **Intervention:** Group A sequence: e-learning/traditional lectures/e-learning; had the same e-learning classroom (storage material) as Group B, 5 min given to study using the computer Group B sequence: Traditional lectures/e-learning/traditional lectures; 5-min discussion with the teacher after the content ended; studied the slides’ content without access to professors for discussion Same content given to Groups A and B simultaneously; after each model, students had 30 min to change to the other classroom	**Written exam:** 7 relevant objectives, 7 questions per module; questions had few words to minimize students’ reading time and increase the test’s reliability; 3 answer choices: true, false or do not know; 126 questions; summative evaluation at end of each module using an objective assessment with 21 questions, same answer choices **Students’ evaluation:** Level of satisfaction with the different teaching methodologies and course content; open-ended questions to gather information about the course, evaluation format and suggestions/criticisms
Fernandez-Lao et al., 2016, [32] Spain, Single-blinded RCT	Under-graduate Physiotherapy students first semester, *n* = 49	**Digital learning design:** Blended learning **Context:** Theoretical acquisition and skills training on campus **Subject/skills:** Musculoskeletal assessment competencies **Duration:** 6 learning lessons and 20 self-study hours **Intervention:** Free access to interactive/app (Ecofisio) as supplement to traditional lectures **Comparison:** In-class, traditional lectures and access to documents and books on the topic	**Written exam:** 20 MCQs, maximum 10 points **OSCE:** Ultrasound and palpation skills assessed; grading system: 3 = excellent, 0 = incorrect; maximum scores: 15 (ultrasound) and 12 (palpation) **Students’ evaluation:** Quality of the intervention; 5-point Likert scale (5 = strongly agree, 1 = strongly disagree); 11-numeric-point rating scale (10 = totally satisfied, 0 = totally unsatisfied)
Huhn et al., 2013, [40] USA, RCT	DPT programme, first year, *n* = 53	**Digital learning design:** Blended learning **Context:** Theoretical acquisition and skills training on campus **Subject/skills:** Pathology II **Duration:** 1 semester **Intervention:** Virtual patient simulation on clinical reasoning, knowledge acquisition, transfer of knowledge and students’ perception of their learning; 6 patient cases; worked individually in campus computer laboratory with the faculty facilitator available only to answer technical questions related to the function of the virtual reality program **Comparison:** In-class, large group discussions; 6 patient cases	**Written exam:** 50 MCQs **Health Science Reasoning Test:** Clinical reasoning prior to and after completing 6 patient cases in their respective group; 30-item test designed to assess induction, deduction, analysis, evaluation and inference skills; overall score and scores for 5 sub-scales **OSCE:** Measure of transfer of learning; observed and scored by a faculty member using a tool developed by the faculty; students graded on professional behaviour and communication, safety, examination, evaluation and interventions using a 5-point scale
Hyland et al., 2010, [41] USA, RCT	Entry-level Physical therapy students, third year, *n* = 33	**Digital learning design:** Distance learning **Context:** Theoretical acquisition on campus **Subject/skills:** Administration and management **Duration:** 1 semester, 9 days **Intervention:** CAI: unlimited access to the course website (Campus Pipeline); received professor’s notes online in a lecture-style format, special examples included within the notes; also received the same PowerPoint presentation, study questions and lecture online as the control group; students could ask questions and share personal experiences via email or online discussion **Comparison:** In-class: PowerPoint presentations; traditional lecture instruction, 4 h per meeting	**Written exam:** Pre- and post-test examination: 25 and 50 MCQs, respectively; score: percentage of questions answered correctly; final course evaluative criteria: final exam (25%), final project (20%), health and wellness assignment (20%), ethics paper (15%) and 2 case studies (10% each)
Lozano-Lozano et al., 2020 [34], Spain, Double-blinded RCT	Under-graduate Physiotherapy Students first and second year, *n* = 110	**Digital learning design:** Blended learning **Context:** Theoretical acquisition and skills training on campus **Subject/skills:** Ultrasound imaging **Duration:** In-class:4 h theoretical lessons and 4 h of practical lessons; self-studies: 2 weeks **Intervention:** In-class: 4 h of theoretical lessons and 4 h of practical lessons; post-class: free access to the Ecofisio interactive website/app **Comparison:** In-class: Two 2-h sessions, traditional lectures; access to books and journal papers on the topic **Both groups:** 2-week self-study period	**OSCE:** Measured participants’ hands-on ultrasound management skills **Written exam:** Evaluation of students’ theoretical knowledge; 20 MCQs; max score: 10 points **Students’ evaluation:** Satisfaction survey with 5-point Likert questionnaire (1 = disagree, 5 = strongly agree); Ecofisio group also completed another satisfaction questionnaire, scores ranged from 0 = totally unsatisfied to 10 = totally satisfied
Maloney et al., 2013, [28] (pilot) Australia, RCT	Under-graduate Physiotherapy students, third year, 2010, *n* = 49	**Digital learning design:** Blended learning **Context:** Skills training on campus **Settings:** Theoretical acquisition and skills training on campus **Subject/skills:** Complex clinical skills **Duration:** First half of students’ third year **Intervention:** 1) 30-min pre-recorded video tutorials: demonstration of the skill, text prompts, trigger and problem solving; 2) Students produced self-video of clinical performance without tutor input or guidance **Comparison:** In-class: traditional teaching with live demonstration of the entire skill; pre-recorded video also shown during practical class with no replay opportunity	**OSCE:** Clinical performance, written patient scenario; grades out of 50 for each performance, 10 set performance criteria: completed well (full marks), partially completed (half marks) or inadequate (zero marks); grades converted to a percentage **Students’ evaluation:** 10-min group-specific survey; questionnaire: perceptions of utility and satisfaction with the teaching methods, 5-point Likert scale (1 = strongly disagree, 5 = strongly agree) and open-ended questions
Maloney et al., 2013, [28] (main) Australia, RCT	Under-graduate Physiotherapy Students, third year, 2009, *n* = 60	**Digital learning design:** Blended learning **Context:** Skills training on campus **Subject/skills:** Clinical skills acquisition **Duration:** 2 weeks **Intervention:** Students created a 5-min self-produced video recording; video reviewed by remote online tutors, often with group feedback on common strengths and weaknesses observed; students reflected on their strengths and areas for improvement; students’ own video clips and the peer benchmark ‘exemplar’ video clip remained online throughout the semester **Comparison:** In-class: clinical skills with regular practical tutoring	**OSCE:** Two clinical skill stations, formative (quantitative and qualitative) feedback to the student on their performance **Students’ evaluation:** Students’ perceptions and experiences: paper-based questionnaire; 5-point Likert scale (1 = strongly disagree, 5 = strongly agree) and open-ended questions
Moore and Smith, 2012, [42] USA, RCT	DPT, Physical therapy students, first year, *n* = 33	**Digital learning design:** Blended learning **Context:** Theoretical acquisition and skills training on campus **Subject/skills:** Psychomotor skills **Duration:** 3 weeks **Intervention:** Video podcasting (videoclips): lecture and podcast demonstrations of transfer skills; students encouraged to review assigned readings and lecture notes and to practice podcast skills; formal class meeting: 2.5 h of lecture and laboratory; students moved directly to the laboratory component of the interaction, beginning with practice and case studies, and utilized the skills depicted in the podcasts in complex patient scenarios **Comparison:** In-class: live instructor demonstration of basic psychomotor skills	**Written exam:** Written post-test on cognitive performance **Practical exam:** Psychomotor performance using a scenario-based practical post-test, graded for safety, fluency and accuracy **Students’ evaluation:** Survey of the 2 learning methods and reported study time; 7 Likert statements and 5 free-response questions
Nicklen et al., 2016, [29] Australia, RCT	Under-graduate Physiotherapy students, third year, *n* = 38	**Digital learning design:** Blended learning **Context:** Theoretical acquisition and skills training on campus **Subject/skills:** Case: Rachel’s pregnancy, the role of the physiotherapist during stages of pregnancy **Duration:** 1 week **Intervention:** Remote-online CBL learning using the same case; web-conferencing with participants physically isolated from one another on campus; WebEx software (written text and audio-visual) **Comparison:** In-class: same case used **Both groups:** Attended the first session (30 min) that introduced key features of interacting via web-conference	**Written exam:** Post-intervention test after second computer session: learning and self-assessed perception of learning, satisfaction and participants’ demographics; 10 MCQs **Students’ evaluation:** Perception of learning measured for each examinable learning objective; 3-point scale: superficial, moderate and in-depth; satisfaction with the remote-online CBL measured on a 5-point scale (1 = strongly disagree, 5 = strongly agree)
Noguera et al., 2013, [33] Spain, Crossover RCT	Under-graduate Physiotherapy students, second year, *n* = 70	**Digital learning design:** Blended learning **Context:** Skills training on campus **Subject/skills**: Practical manual therapy course in a laboratory **Duration:** Two 5-h practical lessons **Intervention:** Anatomy-learning app for mobile devices; Group 1: mobile device used during first practical session; Group 2: mobile device used during second practical session **Comparison:** Description of different manipulative techniques and a practical demonstration performed by the professor; afterwards, students practiced their manipulation technique in pairs (one of them simulating a patient)	**Written exam:** Post-test immediately after each practical session to assess anatomical knowledge; first test: 8 MCQs; second test: 4 open questions and 4 MCQs; score: number of correct answers out of 8 **Students’ evaluation:** Questions 1–17: Likert scale (range 1–5), Questions 19 and 20: Likert scale (range 1–10), Questions 21 and 22: open questions
Rocha et al., 2017, [36] Brazil, RCT	Under-graduate Physiotherapy Students 8th semester *n* = 71	**Digital learning design:** Blended learning **Context:** Theoretical acquisition on campus **Subject/skills:** Professional Practice and Ethics in Physiotherapy discipline **Duration:** Once a week for 17 weeks **Intervention:** Regular classes with extra time for educational video game (quiz type); game room was available until a new room was built with new questions; four formats: the more resources students earned, the more moves they could make **Comparison:** Regular in-person classes	**Written exam:** Specific knowledge test (final exam); 80 questions: single and multiple choice, relationships between columns and true/false **Students’ evaluation:** Satisfaction with the discipline, 5-point Likert scale (1 = not at all satisfied, 5 = very satisfied); perception of learning content, 5-point Likert scale (1 = learned nothing, 5 = learned a lot)
Silva et al., 2012, [37] Brazil, RCT	Under-graduate Physiotherapy students, fourth year, *n* = 16	**Digital learning design:** Blended learning **Context:** Theoretical acquisition on campus **Subject/skills:** Respiratory therapy field **Duration:** 1 semester **Intervention:** Multimodal online environment including multimedia resources (videos, animations and figures) and conventional course classes attended in person; after the end of the course, 2-week access to teachers to ask questions and to the online material to study; access to online material discontinued after 2 weeks, when all students had to take a final exam **Comparison:** In-class: traditional course classes on bronchial hygiene techniques; 2-week access to teachers to ask questions and to online and conventional material to study	**Knowledge test:** 20 questions assessing students’ knowledge of therapeutic indications (8 questions), contraindications for the use of Bronchial Hygiene Techniques (6 questions) and concepts (6 questions); each correct answer scored 0.5 points
Ulrich et al., 2019, [38] Denmark, RCT	Under-graduate Physiotherapy Students, 3 groups: 1: *n* = 28 2: *n* = 26 3: *n* = 27	**Digital learning design:** Blended learning **Context:** Theoretical acquisition and skills training on campus **Subject/skills:** Learning practical skills **Duration:** 1 month **Intervention:** 360° video used as e-learning; after pre-test, Group 1 received lesson using 360° video (Samsung Gear VR), Group 2 received lesson using regular video (laptop) **Comparison:** Group 3 received traditional in-class lesson from an instructor	**Written exam:** Pre-test: MCQs on the learning requirements for the treatments **Practical exam** post-test: after treatment, tested on learning, practical setting: patient (volunteer) and a teacher in physiotherapy education recorded the results; graded: pass/fail for each question or task **Students’ evaluation:** Questionnaire about students’ learning satisfaction and perception of the learning climate in each treatment group (given after final test)
Covill and Cook, 2019, [43] USA, Comparative cohort study	DPT, Physiotherapy first year, 3 classes: A: *n* = 47 B: *n* = 54 C: n = 47	**Digital learning design:** Flipped classroom **Context:** Theoretical acquisition **Subject/skills:** Musculoskeletal content, patient management of the lower quadrant **Duration:** 81 lecture hours and 79 laboratory hours **Intervention:** Classes B and C: flipped classroom (alternating lecture hours); pre-class: pre-recorded lectures, readings, non-graded quizzes and discussion questions; in-class: faculty-led large group question and case discussion, small group question and case discussion, polling software and quiz discussion; **Comparison:** Class A: 18 h of traditional lectures and 31 h of laboratory work	**Written exam:** 10 tests total, delivered every 2 weeks; 83 MCQs across all 3 cohorts specific to the content delivered **Students’ evaluation:** Classes B and C (flipped classroom) received a post-course survey specific to student perceptions of the flipped method; 5-point Likert scale
Day, 2018, [44] USA, Cohort	DPT, Physical therapy first two semesters, *n* = 112	**Digital learning design:** Flipped classroom **Context:** Theoretical acquisition and skills training on campus **Subject/skills:** Gross anatomy course **Duration:** 15 week-long courses **Intervention:** Flipped classroom; pre-class: 15-min instructor-created lecture videos prior to class (less than 60 min per week); in-class: 130 min/week, included the same activities from previous year; students also participated in a prosected cadaver laboratory 90 min per week **Comparison:** Traditional in-class lectures and prosected cadaver laboratory for 90 min per week × 15 weeks	**Written exam:** 120 MCQs. All final examination MCQs were divided into two levels. Lower-level MCQs (LL-MCQ) were define as “remember” and “understand” and included questions that required recall of definitions and terms. Higher-level MCQs (HL-MCQ) were defined as “apply”and “analyse.” These questions required participants to use higher-order cognitive skills to apply knowledge to new situations. No items were “create” or “evaluate,” due to the nature of the MCQ examination. In total, 13 final examination MCQs were determined to be at a higher cognitive domain; apply or analyse. The HL-MCQs included anatomical identification on MRI images and clinical scenarios that required students to analyse the facts of the case to determine the location of an injury or possible symptoms present. During the subsequent kinesiology course, students received 3 MCQ unit examinations that remained consistent between the two groups; traditional and flipped classroom format. Each examination was not cumulative, and no final examination was given. Student’s kinesiology grades from each of the examinations and the overall semester grade was obtained from the instructor of record.
Deprey, 2018, [45] USA, Cohort	Under-graduate Physiotherapy students, fifth year, 3 groups: 1: *n* = 44 2: *n* = 49 3: *n* = 50	**Digital learning design:** Flipped classroom **Context:** Theoretical acquisition and skills training on campus **Subject/skills:** Neurological disorders **Duration:** 2-h time blocks, 3 days per week **Intervention 1:** Fully integrated flipped; pre-class: 5 pre-recorded lectures, in-class: worked in groups to answer instructor-posed questions and complete scenarios; internet searches or open book or note reviews; focus: student questions **Intervention 2:** Partially integrated flipped; pre-class: recorded lectures, in-class: reiteration of recorded lectures and discussion without special in-class work, opportunity to ask questions or clarify concepts Both: 2-h balance test and measures lab **Comparison:** Five 2-h in-class lectures, individual homework and 2-h balance test and measures lab	**Written exam:** Given at completion of each of the 3 units; exams 1 and 2 included the same items for all 3 years of the study; exam scores assessed for objective change in content knowledge; primary outcome: scores on the second unit exam; changes in scores from exam 1 to exam 2 were compared
Green and Whitburn, 2016, [26] Australia, Retrospective cohort	Under-graduate Physiotherapy students, second year, 3 groups: 1: *n* = 150 2: *n* = 160 3: *n* = 151	**Digital learning design:** Blended learning **Context:** Theoretical acquisition and skills training on campus **Subject/skills:** Gross anatomy **Duration:** 15 week-long courses **Intervention:** Group 3: fully blended; pre- and in-class: online video clips, face-to-face lectures, practical classes, clinical anatomy classes, face-to-face tutorials **Comparison:** Group 1: in-class, traditional lectures; Group 2: in-class lectures and some online content (video clips)	**Practical and written exam:** Aggregate practical test mark (expressed as a percentage to avoid differences in weighting between cohorts) and final written examination mark (expressed as percentage) between the cohorts **Students’ evaluation:** Questionnaire, 5-point Likert scale (5 = strongly agree, 1 = strongly disagree), open-ended questions
Murray et al., 2014, [46] USA, Cohort	Under-graduate Physiotherapy students, third semester, 2 groups: 1: *n* = 43 2: *n* = 35	**Digital learning design:** Flipped classroom **Context:** Theoretical acquisition and skills training on campus **Subject/skills:** Pathological conditions of the extremities **Duration:** 1 semester **Intervention:** Flipped classroom; pre-class: 10 to 25-min asynchronous online lectures in SAKAI (course management system), students encouraged to take notes and bring questions to class for discussion; face-to-face in-class meeting: 15 min to clarify any information that was unclear from online lectures, 20 to 30-min PowerPoint presentation integrating the online lecture content into examination sequence, 120 to 240-min group discussions of cases with emphasis on clinical decision making **Comparison:** Traditional face-to-face lectures	**Final exam:** 105 MCQs; correct answers tallied in aggregate and by cohort based on 5 areas: [1] total exam score, [2] score on examination/evaluation questions, [3] score on intervention questions, [4] score on lower-level questions and [5] score on higher-level questions

*RCT* Randomized controlled trial, *DPT* Doctor of Physical Therapy, *MCQ* Multiple choice question, *OSCE* Objective structured clinical evaluation, *App* Application, *CAI* Computer-assisted instruction, *CBL* Case-based learning

*DPT* Doctor of Physical Therapy; *MCQ* Multiple choice question

### Description of interventions

All the included studies compared digital learning designs to traditional classroom teaching. The duration of exposure to the digital learning designs ranged from 10 h to two semesters. In the blended learning designs, 21 studies used different digital learning technology and software—such as interactive websites/apps, multimodal online environments (e.g., videos, animations and figures), recorded videos/lectures/tutorials, simulation learning videos with virtual cases/scenarios, video clips (video podcasting) and educational videogames—to present and facilitate the learning materials and to assess the learning outcomes on practical skills and/or knowledge acquisition [25–40, 42–46].

Only one study used the distance learning design [41]. In this study, an interactive course website (i.e. CAI) was used to facilitate learning. Students had unlimited access to the course website. All the course content and learning activities were facilitated as asynchronous learning, and there was no face-to-face teaching.

The pre-class and in-class digital activities integrated different learning activities in the blended learning and distance learning designs. These learning activities were facilitated asynchronously (flexible time and distance) and/or synchronously (in real time; either distanced or in a classroom or laboratory). The four blended learning design studies that utilized flipped classrooms expected the students to be prepared by completing pre-class activities (asynchronous online learning) before in-class teaching [43–46]. Examples of pre-class activities were pre-recorded lessons and different tasks to achieve knowledge acquisition by listening, reading and/or observation. None of these studies described pre-class collaborative learning activities using digital learning tools or digital learning resources, but rather facilitated different collaborative learning in-class activities (e.g., group questions and case discussions, polling software and quiz discussions).

In the other blended learning designs, in-class activities required students to listen to or observe the teacher/tutor as well as conduct observations in the classroom and/or practice (i.e. in a laboratory or clinical immersion setting). For clinical immersion, the simulation learning activities [25] included time-outs, rewinds, debriefing and reflection sessions with a clinical educator. Another study with a blended learning design involved an e-learning classroom of storage material [35]. The intervention was a sequence of traditional/e-learning/traditional classroom designs and e-learning/traditional/e-learning designs. For more information on the characteristics of the included studies, see Table 2.

### Risk of bias assessment

**Fig. 2:**
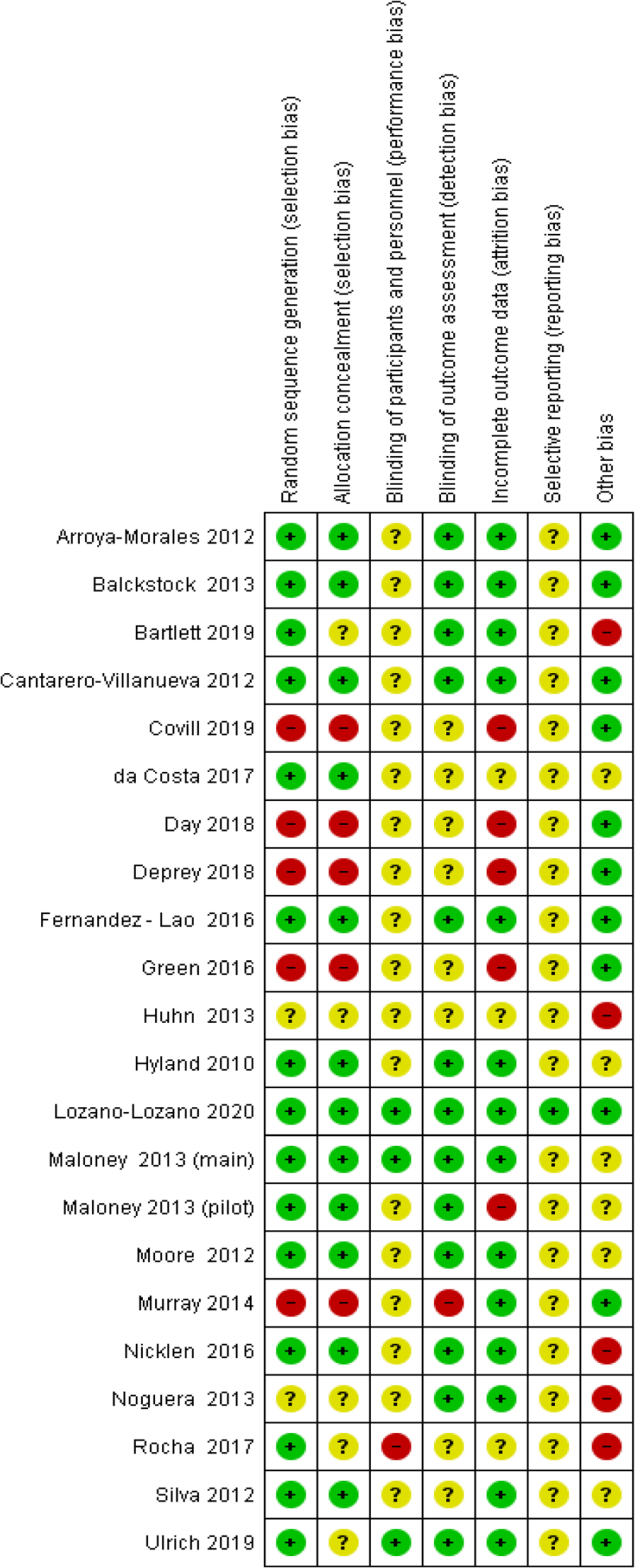
Risk of bias summary: review authors’ judgements about each risk of bias item for each included study

We determined that the overall risk of bias was higher for the cohort studies [26, 43–46] than the RCTs [25, 27–42], (Fig. 2). The cohort’s studies had a high risk of selection bias and attrition bias. Additionally, domains such as blinding and selective reporting were poorly described in the cohort’s, and therefore the risk was unclear.

The RCTs [25, 27–42], had a low or unclear risk of bias in the domains of performance bias, detection bias and reporting bias (Fig. 2). It was not possible to blind the students to the digital learning design interventions. Therefore, we assessed the domain of performance bias as unclear.

### Effects of blended learning designs using flipped classroom on knowledge acquisition

**Fig. 3:**
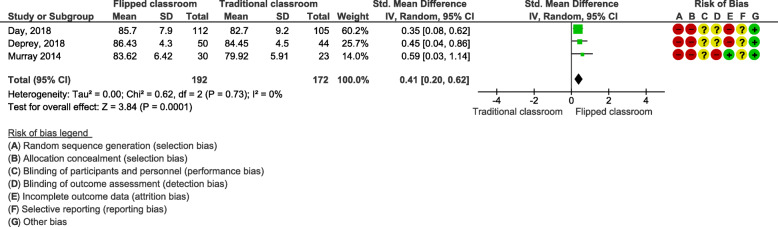
Flipped classroom as blended learning designs on knowledge acquisition assessed by MCQ

We conducted a meta-analysis of the effects of flipped classrooms compared to traditional classroom teaching on knowledge acquisition graded using multiple-choice questions (MCQs). See Additional File 2, Table 2 for more details. Three cohort studies were included in this meta-analysis for a total of 364 students [44–46]. The meta-analysis showed a SMD of 0.41 (95% CI: 0.20, 0.62; Fig. 3). This result was statistically significant and implied that students who participated in a flipped classroom earned higher grades/scores on the MCQs than students who were enrolled in a traditional classroom (Fig. 3).

#### Effects of additional study using flipped classroom

Another study using flipped classroom interventions could not be included in the meta-analysis because of poor reporting of effect estimates [43]. This study included 148 students, and the result showed high correlation with similar performance between all classes.

### Effects of blended learning designs using interactive websites/apps on knowledge acquisition

**Fig. 4:**
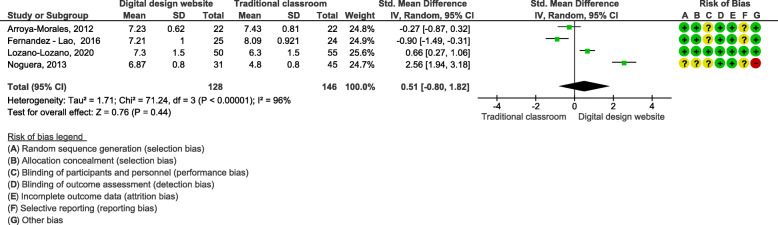
Blended learning designs using interactive websites/apps on knowledge acquisition assessed by MCQ

We pooled four studies (*n* = 279 students) that used interactive websites/apps in their blended digital learning designs and compared them to traditional classroom teaching on knowledge acquisition assessed by MCQs [30, 32–34]. The meta-analysis showed a SMD of 0.51 (95% CI: − 0.80, 1.82; with an I^2^ of 96%, Fig. 4). This result showed no statistically significant difference between blended learning and traditional classroom teaching on knowledge acquisition.

### Effects of blended learning designs using interactive website/app on practical skills

**Fig. 5:**
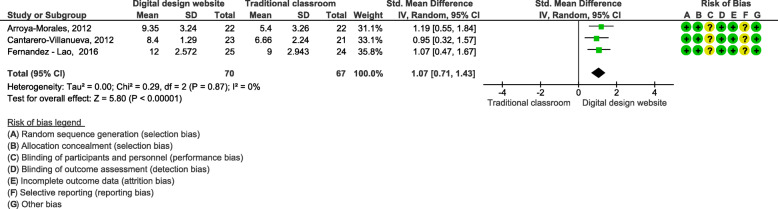
Blended learning designs using interactive website/app on practical skills assessed by OSCE

Three studies used the same interactive website/app (Ecofisio) to teach practical skills, which were assessed by objective structured clinical evaluation (OSCE) [30–32]. These studies included 137 students in total. The meta-analysis showed a SMD of 1.07 (95% CI: 0.71, 1.43; Fig. 5) and a statistically significant difference in favour of the blended learning design.

#### The effects of additional studies in blended learning designs using mobile applications

Another blended learning study with 110 students also investigated the effect of the same interactive website/app on practical skills assessed by OSCE [34]. While the results indicated significant differences for all components assessed using OSCE, the results were poorly reported and thus could not be pooled with the others in the meta-analysis. Additionally, another study was not included in this meta-analysis due to use of a different mobile application [39]. This application included videos and written content but was not interactive, and the study tested a different outcome, a practical exam. This study included 20 students distributed in three groups: the control group (demonstration only), the mobile application and demonstration group and the mobile application only group. The primary competency—the ability to perform and explain clinical skills—was highest among the demonstration plus app group followed by the demonstration only group and finally the app only group. This was consistent with the results of the above meta-analysis regarding the effect of the interactive website/app on practical skills.

### Effects of blended learning designs using self-produced videos on practical skills

**Fig. 6:**
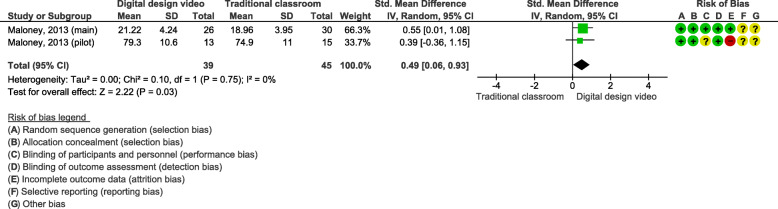
Blended learning designs using self-produced videos on a practical skill in a cervical spine scenario assessed by OSCE

**Fig. 7:**
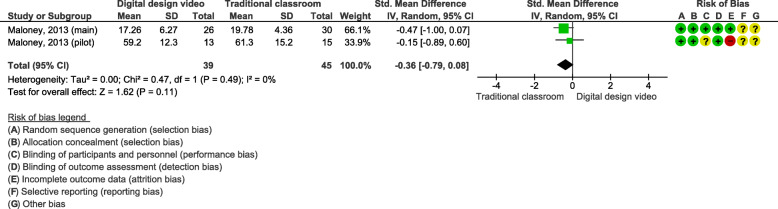
Blended learning designs using self-produced videos on a practical skill in a vestibular implant scenario assessed by OSCE

Two studies (*n* = 84 students) assessed self-produced videos on OSCE [27, 28]. These interventions also included pre-recorded video tutorials with demonstrations of the skill. The outcomes were tested for practical skills in a cervical spine scenario (Fig. 6) and a vestibular implant scenario (Fig. 7).

The meta-analysis for a practical skill in the cervical spine scenario showed a SMD of 0.49 (95% CI 0.06, 0.93 Fig. 6). There was a statistically significant difference between the groups’ final exam scores for the cervical spine scenario.

The meta-analysis for a practical skill in the vestibular implant scenario showed a SMD of − 0.36 (95% CI: − 0.79, 0.08; Fig. 7). No significant differences were observed between the blended learning design and traditional classroom teaching for the vestibular implant scenario.

### The effects of additional blended learning designs using other video formats and outcome

Three blended learning design studies using video formats were not included in the above meta-analysis [25, 26, 42]. This was due to their use of different interventions or outcomes compared to the studies that were included in that meta-analysis. For example, one study with 33 students investigated the effect of using video clips on practical exam scores [42]. The results showed no statistically significant difference compared to traditional classroom teaching. This was in line with the results of the meta-analysis regarding the effects of self-produced videos for a practical skill in the vestibular implant scenario.

Another study with 461 students incorporated online video clips (video podcasting) and asynchronous online discussion forums and tested their effects on practical and written exam scores [26]. This study showed statistically significant differences in scores in using the online video clips and online discussion forums compared to traditional classroom teaching. This was in line with the results of the meta-analysis regarding the effects of self-produced videos for a practical skill in the cervical spine scenario.

Finally, a study of 349 students investigated the effect of simulated learning environment videos on practical exam scores [25]. This single-blinded, multi-institutional RCT study showed no significant improvement in student competency. This result is consistent with the previous meta-analysis regarding meta-analysis on blended learning designs using self-produced videos for a practical skill in the vestibular implant scenario.

### Effects of blended learning designs on students’ perceptions of learning

**Fig. 8:**
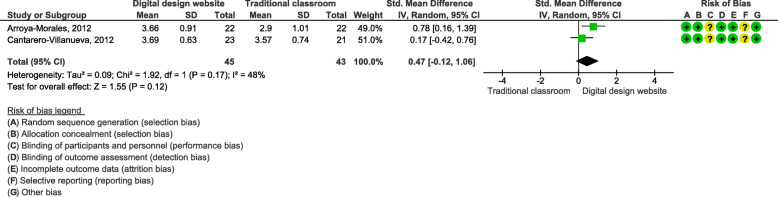
Students’ learning perceptions; Item: “I was able to apply what I learned”

Two studies assessed students’ perceptions of learning using an interactive website/app [30, 31]. We focused on the item ‘I was able to apply what I learned’. These studies included 83 students and used a Likert scale of 1–5 (1 = strongly disagree, 5 = strongly agree). The meta-analysis showed a SMD of 0.47 (95% CI: − 0.12, 1.06; Fig. 8), but the results was not statistically significant.

#### The effects additional blended learning designs on students’ learning perceptions

Thirteen studies that were not included in that meta-analysis assessed students’ perceptions of blended learning designs using various evaluation items [25, 26, 28, 29, 32–36, 38, 42, 43]. See Additional File 2, Table 2. One study showed higher satisfaction levels in the intervention group (interactive website/app) for the item ‘I believe that training was applicable’ [34].

Another study assessed the effects of remote-online case-based learning (CBL) [29] on students’ self-assessed perception of learning for each examinable objective using a 3-point scale with the options of ‘superficial’, ‘moderate’ and ‘in depth’. For the item ‘I felt I was able to achieve all objectives given the method of CBL delivery’, 12 out of 19 participants in the intervention group disagreed with the statement.

Overall, for seven of the 13 studies [26, 28, 32, 33, 36, 43] that were not included in the meta-analysis on students’ perceptions of learning, statistically significant results and higher perceptions of learning were found in the intervention groups. The results from all the studies that evaluated students’ perceptions of learning are available in Additional File 2, Table 2.

#### The effects of additional interventions using blended learning designs

Six of the blended design studies [29, 35–38, 40] used different digital learning technologies and/or outcomes from the studies included in the meta-analyses [27, 28, 30–34, 44–46]. See Additional File 2, Table 2. Of the blended learning designs, one study used a multimodal online environment (videos, animations and figures) and was assessed by a knowledge test [37]. The study included 16 students, and the outcome was theoretical knowledge acquisition. The results showed a significant improvement in acquisition among the students who participated in the multimodal online environment compared to the students in the control group.

A second blended learning design study used e-lectures, and a knowledge test to assess the effect on theoretical acquisition [35]. This study included 72 students. The results showed significant improvement in theoretical acquisition among the students who viewed the e-lectures compared to those who observed traditional classroom teaching.

A third blended learning design study used an educational video game, and students’ resulting theoretical acquisition was assessed by a knowledge test [36]. This study included 71 students, and the results showed that the educational video game was able to improve performance on the specific knowledge test.

A fourth blended learning design study used 360° video as the e-learning tool, and the outcome, theoretical acquisition, was assessed by MCQ [38]. This study included 81 students. The findings indicated that there was no significant difference between 360° video and traditional teaching.

A fifth blended learning design study used virtual patient simulation [40]. The outcomes were theoretical acquisition and practical skills and were assessed by MCQ. This study included 53 students. The researchers found no significant differences between the Health Science Reasoning Test scores based on the method of instruction.

Finally, a sixth blended learning design study with 38 students used web conferencing remote-online CBL [29]. The outcome, theoretical knowledge acquisition, was assessed by MCQ. Of the 15 examinable learning objectives, eight were significant in favour of the control group, suggesting a greater perceived depth of learning for the students in the control group.

#### The effects of additional intervention using distance learning design on knowledge acquisition

One study assessed the effects of a distance learning design using a course website (CAI) as an intervention [41]. This study included 33 students. The results showed no significant differences between the groups for baseline knowledge; see Additional File 2, Table 2.

## Discussion

The aim of this systematic review was to identify and investigate the effectiveness of digital learning designs in physiotherapy education. The main findings are that all except one included study (21 out of 22) applied a blended learning design. Out of these 21 studies, 19 studies showed equal or statistically significant differences in favour of blended learning compared to traditional classroom teaching.

Among the blended learning designs, flipped classroom was the most frequently identified approach. Notably, in terms of effectiveness, the meta-analysis showed a statistically significant improvement in learning outcomes for the flipped classroom designs [44–46]. These findings are in line with another systematic review of 12 studies that showed significant improvement in students’ self-directed learning skills in nursing education [47]. In contrast, findings in a review of 24 studies in health professions education concluded with no clear evidence that the flipped classroom produced better academic outcomes [6]. The pedagogical opportunities offered by the flipped classroom model have the potential to motivate and engage students in pre-class learning activities, enhance self-regulative abilities among students and improve the flexibility and transparency of the learning process [48]. Further, in-class activities require active students and enhanced opportunity to apply new content to a prior knowledge to solve problems and may led to higher order thinking. Another opportunity is to receive feedback from peers and teachers in real time [49]. Thus, these pedagogical possibilities can lead us to conclude that the flipped classroom model is promising in terms of enhancing students’ learning outcomes [48].

The effect estimates of using an interactive website/app on practical skills showed statistically significant benefits of the interactive website/app [30–32]. This is supported by another systematic review that included 29 studies, which indicated that mobile learning is as effective as or possibly more effective than traditional learning [50]. There are several possible explanations for the results of our meta-analysis on the use of interactive websites/apps on practical skills [30–32]. Interactive websites/apps are flexible, accessible and transparency and allow students to observe how to perform practical skills and to acquire theoretical knowledge. In general, research also shows that the use of mobile learning technology in higher education courses increases enjoyment, attention and learning [51].

It has been claimed that implementation of mobile learning is a challenging endeavor and some of the most demanding aspects of mobile learning ‘are the links between and the need to facilitate different sustainable pedagogical and learning strategies by integration, support, interactive use and appropriate choice of tools’ [4] (p.32). Mobile leaning is promoted when the applications focus on students’ newly acquired knowledge and skills [49]. In the three studies in this meta-analysis, students in the intervention group were given free access to the interactive website/app immediately after the traditional classroom teaching was finished [30–32], which may explain their effectiveness. Another explanation for the significant differences between the interactive websites/apps and traditional learning resources is that the interactive design of the mobile learning activities were in line with the learning outcomes and type of assessment method [52]. Further, interactive websites/apps can support and facilitate ‘authentic learning (tasks related to the learning outcomes), situated learning (takes place in the surroundings applicable to the learning) and facilitate context-aware learning (history and the environment) due to its affordances, accessibility, portability, and educational benefits’ [53] (p. 2).

The behaviourist learning approach with teachers acting as content deliverers is often used in mobile learning designs in higher education [4]. From a critical perspective, apps must be integrated into the learning system for different learning materials (e.g., books and articles), and the content, learning activities and technology must be designed in such a way that the activities (interactive) and technology complement each other, which will support students to achieve the learning outcomes [4].

One meta-analysis showed statistically significant improvement of self-produced videos compared to traditional classroom teaching on a practical skill in a cervical spine scenario [27, 28]. Due to few included participants this result needs to be confirmed in a larger meta-analysis. Combining practical classroom teaching and students self-produced video performing practical skills, might promote higher skills acquisition, compared to practical classroom teaching alone. An explanation of this effect is the ability to connect knowledge that has being transferred to practical implications and student’s performance. This is in line with mobile learning when the applications focus on students’ newly acquired knowledge and skills [49]. Using self-produced videos as a supplement to the practical classroom teaching also give the teacher/tutors/supervisors an opportunity to provide students with feedback on their clinical performance. Further, self-produced videos give the ability for peer-to-peer learning by sharing and discussion the results in the self-produced videos and the possibility to self-reflections in the process of developing professional clinical skills.

Thirteen studies that were not included in this meta-analysis also assessed students’ perceptions of blended learning designs using different evaluation items [25, 26, 28, 29, 32–36, 38, 42, 43]. Of these 13 studies, 7 studies showed that students had a positive experience and significantly higher perceptions of learning with the blended learning designs [26, 28, 32–34, 36, 43]. An explanation for this is that the blended learning design has the potential to facilitate and support students’ self-directed learning, independence, intrinsic motivation and responsibility [53]. Another explanation is that these blended learning designs probably had a planned didactic learning design that integrated digital learning technology and had a constructive alignment approach. Thanks to these characteristics, the blended and distance learning designs that were not included in the meta-analyses overall seemed to improve students’ academic performances (e.g. grades) or at least as equally effective as traditional classroom teaching. These findings are in line with other studies demonstrating increased student involvement, engagement, communication, critical discussions, and student–teacher contact [13, 54]. However, there is a criticism to technology optimism promoted by Fossland and Ramberg [55] ongoing that there is an uncritical belief that the use of technology leads to learning in itself. In line with this criticism Lillejord et al. [4] stated that how digital tools are implemented and used pedagogically, rather than the technology itself, is what affects students’ learning outcomes.

The present review had similarities with a systematic review from 2015 by Mącznik et al. on online technology use in physiotherapy teaching and findings in both reviews indicate that digital learning designs offer benefits for teaching and learning in physiotherapy education [13]. There are, however, some differences that should be noted: first, the present review exclusively investigated the effectiveness of digital learning designs, while the review by Mącznik et al. additionally investigated users’ perception [13]. Due to this, only studies with summative assessments for the final exam, was included in our review. Second, the present review had a broader approach and included all types of digital learning designs, not only online technologies. In addition, it is worth noting that the present review includes a number of recent studies, thus presenting an up-to-date picture of the digital learning designs.

### Strengths and limitations

This systematic review has two main strengths. First, two of the authors (N.BØ, Y.R), together with two information specialists at the Oslo Metropolitan University (M.WG, E.K), developed a rigorous and comprehensive search strategy on digital learning technology in learning design. Second, we were able to synthesise the studies and conduct meta-analyses even though the included studies had different interventions, small sample sizes and varied effects sizes.

However, this review has some limitations. First, several of the included studies had weak study designs (single cohorts), underreported statistical methods and educational intervention details or used non-validated outcome measurement methods (e.g., MCQs and self-report questionnaires). It was also difficult to accurately assess the risk of bias for some of the included studies due to poorly reported studies. Only one of the included studies had a long-term (two-semester) follow-up to assess learning retention. Finally, the included studies used various conceptions of blended and distance learning designs. This generated an unclear terminology and made it difficult to compare designs and synthesize the results.

### Recommendations

More robust studies, such as experimental designs, are needed for this topic. Additionally, future studies need to incorporate control variables and statistical methods for reporting the results, especially those using flipped classroom designs. More in-depth and follow-up research studies assessing learning retention, students’ approaches to learning and studying in a digital learning environment would also be beneficial. Furthermore, scholars should investigate the experiences and attitudes of teachers towards developing and implementing digital learning designs in physiotherapy education.

## Conclusions

This systematic review identified blended learning and distance learning designs in physiotherapy education. The results indicated that blended learning designs tend to be either equally or more effective as traditional classroom teaching in physiotherapy education in terms of knowledge- and practical skills acquisition. In contrast, the results for the one distance learning design demonstrated equally results compared to traditional classroom teaching.

The meta-analyses revealed significant effects on student learning in favour of the interventions using flipped classrooms, interactive websites/apps and students self-produced videos. However, these results need to be confirmed in larger controlled trials. Additionally, the generalization of this finding is limited to the physiotherapy population studied in this review. This review highlights the need for improvements in future studies’ methodological designs.

## Supplementary Information

**Additional file 1.**

**Additional file 2.**

## Data Availability

The datasets used and/or analysed for this study are available from the corresponding author upon reasonable request.

## Abbreviations

CAI: Computer-assisted instruction
App: Mobile application
CI: Confidence interval
RCT: Randomized controlled trial
SMD: Standardized mean difference
MCQ: Multiple-choice question
OSCE: Objective structured clinical evaluation
CBL: Case-based learning
DPT: Doctor of Physical Therapy
PhD: Doctor of Philosophy
