# Supplementary material for: Digital learning designs in physiotherapy education: a systematic review and meta-analysis
Source: BMC Med Educ. 2021 Jan 13;21:48. doi: 10.1186/s12909-020-02483-w (PMC7805166; doi:10.1186/s12909-020-02483-w)
Supplement: Supplementary file 1 — Additional file 1. [file 12909_2020_2483_MOESM1_ESM.pdf]

Appendix 1

*Search strategy and preliminary search results: Ovid MEDLINE(R) and Epub Ahead of Print

Ovid MEDLINE(R) and Epub Ahead of Print, In-Process and Other Non-Indexed Citations, daily, 1946 to April 03, 2019		
#	Searches	Results
1	Physical Therapists/	1,432
2	Allied Health Personnel/	11,326
3	Occupational Therapists/	158
4	(physiotherap* or physical therap* or allied health or occupational therap*).tw,kw,kf.	62,505
5	1 or 2 or 3 or 4	72,601
6	education/ or exp education, professional/ or education, continuing/ or exp education, graduate/ or mentoring/ or preceptorship/ or exp schools/ or exp educational measurement/ or Teaching/ or Curriculum/	488,670
7	exp Students/	116,243
8	education.fs.	263,932
9	6 or 7 or 8	631,564
10	5 and 9	10,532
11	((physiotherap* or physical therap* or allied health or occupational therap*) adj3 educat*).tw,kw,kf.	2,237
12	((physiotherap* or physical therap* or allied health or occupational therap*) and (school* or student*)).tw,kw,kf.	5,292
13	10 or 11 or 12	13,591
14	Computer Assisted Instruction/	11,437
15	Education, Distance/	3,552
16	Educational Technology/	1,421
17	Webcasts as Topic/	303
18	Information Technology/	193
19	Computers, handheld/	3,329
20	Smartphone/	2,769
21	Wireless technologies/	3,035
22	Multimedia/	1,830
23	(blended or e learning or elearning or m learning or mlearning or webbased or web based or virtual* or streaming or interactiv* or hybrid or digital* or gami* or game* or ict or mooc or massive open online course* or flipped or simulation*).tw,kw,kf.	807,773
24	(online adj (learning or syllabus or activit* or teaching or educat* or com* or tech* or network* or discussion* or participat*)).tw,kw,kf.	5,022
25	(blog* or wiki* or podcast* or webcast* or courseware* or groupware* or collaborative software* or webinar* or "web 2.0" or smartphone* or lecture capture* or tablet* or ((Computer* or device*) adj1 handheld)).tw,kw,kf.	62,391
26	(comput* adj (based or assisted or aided or supported or use* or instruction* or supported or educat*)).tw,kw,kf.	57,518

27	(“computers and learning” or “computers in education” or (audience adj3 respon*)).tw,kw,kf.	474
28	((electronic or mobile or distance or multimedia or asynchronous or computer*) adj2 (learning or educat*)).tw,kw,kf.	5,089
29	((web or technolog* or internet) adj1 (based or enhanced or enabled or mediated)).tw,kw,kf.	42,577
30	(instructional television* or information communication or intelligent tutoring system*).tw,kw,kf.	718
31	(((education* or learning) adj1 (media* or multimedia*)) or computerized instruction or mobile device*).tw,kw,kf.	3,943
32	((information or education* or instruction* or use*) adj2 tech*).tw,kw,kf.	135,021
33	or/14-32	1,063,260
34	13 and 33	1,158
35	limit 34 to yr= “2010-Current”	728

	Ovid MEDLINE(R) and Epub Ahead of Print, In-Process & Other Non-Indexed Citations and Daily 1946 to February 24, 2020	
#	Searches	Results
1	Physical Therapists/	1715
2	Allied Health Personnel/	11617
3	Occupational Therapists/	259
4	(physiotherap* or physical therap* or allied health or occupational therap*).tw,kw,kf.	67237
5	or/1-4	77597
6	education/ or exp education, professional/ or education, continuing/ or exp education, graduate/ or exp schools/ or exp educational measurement/ or Teaching/ or Curriculum/	508050
7	exp Students/	124652
8	education.fs.	273930
9	or/6-8	656883
10	5 and 9	11025
11	((physiotherap* or physical therap* or allied health or occupational therap*) adj3 educat*).tw,kw,kf.	2387
12	((physiotherap* or physical therap* or allied health or occupational therap*) and (school* or student* or curriculum)).tw,kw,kf.	5946
13	or/10-12	14374
14	Computer Assisted Instruction/	11719

15	Education, Distance/	3832
16	Educational Technology/	1500
17	Webcasts as Topic/	323
18	Information Technology/	307
19	Computers, handheld/	3522
20	Smartphone/	3912
21	Wireless technologies/	3370
22	Multimedia/	1904
23	(blended or e learning or elearning or m learning or mlearning or webbased or web based or virtual* or streaming or interactiv* or hybrid or digital* or gami* or game* or ict or mooc or massive open online course* or flipped or simulation*).tw,kw,kf.	871589
24	(online adj (learning or syllabus or activit* or teaching or educat* or com* or tech* or network* or discussion* or participat*)).tw,kw,kf.	5666
25	(blog* or wiki* or podcast* or webcast* or courseware* or groupware* or collaborative software* or webinar* or "web 2.0" or smartphone* or lecture capture* or tablet* or ((Computer* or device*) adj1 handheld)).tw,kw,kf.	67732
26	(comput* adj (based or assisted or aided or supported or use* or instruction* or supported or educat*)).tw,kw,kf.	60739
27	("computers and learning" or "computers in education" or (audience adj3 respon*)).tw,kw,kf.	510
28	((electronic or mobile or distance or multimedia or asynchronous or computer*) adj2 (learning or educat*)).tw,kw,kf.	5470
29	((web or technolog* or internet) adj1 (based or enhanced or enabled or mediated)).tw,kw,kf.	46746
30	(instructional television* or information communication or intelligent tutoring system*).tw,kw,kf.	806
31	(((education* or learning) adj1 (media* or multimedia*)) or computerized instruction or mobile device*).tw,kw,kf.	4438
32	((information or education* or instruction* or use* or using) adj2 tech*).tw,kw,kf.	224950
33	or/14-32	1218259
34	13 and 33	1310
35	limit 34 to (yr="2019 -Current" and (danish or english or norwegian or swedish))	140

	Ovid MEDLINE(R) and Epub Ahead of Print, In-Process & Other Non-Indexed Citations and Daily 1946 to August 27, 2020	
#	Searches	Results
1	Physical Therapists/	1923
2	Allied Health Personnel/	11760
3	Occupational Therapists/	310
4	(physiotherap* or physical therap* or allied health or occupational therap*).tw,kw,kf.	70286
5	or/1-4	80791
6	education/ or exp education, professional/ or education, continuing/ or exp education, graduate/ or exp schools/ or exp educational measurement/ or Teaching/ or Curriculum/	518573
7	exp Students/	129292
8	education.fs.	279246
9	or/6-8	670679
10	5 and 9	11294
11	((physiotherap* or physical therap* or allied health or occupational therap*) adj3 educat*).tw,kw,kf.	2498
12	((physiotherap* or physical therap* or allied health or occupational therap*) and (school* or student* or curriculum)).tw,kw,kf.	6238
13	or/10-12	14846
14	Computer Assisted Instruction/	11880
15	Education, Distance/	4111
16	Educational Technology/	1521
17	Webcasts as Topic/	342
18	Information Technology/	386
19	Computers, handheld/	3615
20	Smartphone/	4548
21	Wireless technologies/	3587
22	Multimedia/	1930
23	(blended or e learning or elearning or m learning or mlearning or webbased or web based or virtual* or streaming or interactiv* or hybrid or digital* or gami* or game* or ict or mooc or massive open online course* or flipped or simulation*).tw,kw,kf.	915251

24	(online adj (learning or syllabus or activit* or teaching or educat* or com* or tech* or network* or discussion* or participat*)).tw,kw,kf.	6263
25	(blog* or wiki* or podcast* or webcast* or courseware* or groupware* or collaborative software* or webinar* or "web 2.0" or smartphone* or lecture capture* or tablet* or ((Computer* or device*) adj1 handheld)).tw,kw,kf.	71563
26	(comput* adj (based or assisted or aided or supported or use* or instruction* or supported or educat*)).tw,kw,kf.	62756
27	("computers and learning" or "computers in education" or (audience adj3 respon*)).tw,kw,kf.	529
28	((electronic or mobile or distance or multimedia or asynchronous or computer*) adj2 (learning or educat*)).tw,kw,kf.	5838
29	((web or technolog* or internet) adj1 (based or enhanced or enabled or mediated)).tw,kw,kf.	49676
30	(instructional television* or information communication or intelligent tutoring system*).tw,kw,kf.	852
31	(((education* or learning) adj1 (media* or multimedia*)) or computerized instruction or mobile device*).tw,kw,kf.	4818
32	((information or education* or instruction* or use* or using) adj2 tech*).tw,kw,kf.	232818
33	or/14-32	1274857
34	13 and 33	1400
35	limit 34 to (yr="2020 -Current" and (danish or english or norwegian or swedish))	85
