# Supplementary material for: Digital learning designs in physiotherapy education: a systematic review and meta-analysis
Source: BMC Med Educ. 2021 Jan 13;21:48. doi: 10.1186/s12909-020-02483-w (PMC7805166; doi:10.1186/s12909-020-02483-w)
Supplement: Supplementary file 2 — Additional file 2. [file 12909_2020_2483_MOESM2_ESM.docx]

**Additional file 2**

**Table 3 – results from all included studies**

| **Author, year**  **Risk of bias** | **Digital learning design and intervention** | **Results** |
| --- | --- | --- |
| Arroyo Morales, et.al., 2012  Low | Blended learning  Interactive website/app | **MCQ** No significant intergroup difference in the acquisition of theoretical knowledge as assessed by the MCQ (*p* =.390)  **OSCE** Scores were significantly higher in the experimental group than in the control group for skills in palpation ability and ultrasound assessment of the knee. Students in the e-learning group needed less time to palpate the musculoskeletal structure (*p* = .041), but more time (*p* = .012) to obtain their superior ultrasound images.  **Evaluation** Students in both groups were interested in learning another anatomical region, higher interest in the control group. Neither group would have preferred to be in the other group in the study. Experimental group reported a high level of satisfaction with the characteristics of the website. |
| Bartlett and Smith,  2020  Unclear | Blended learning  Mobile application (app) | **Practical exam** Total competency, the ability to perform and explain clinical skills was highest among the demonstration and app group (13.33 ±3.07), followed by the demonstration only group (11.14±2.47), and finally the app only group (8.14±4.18). 5.1 (95% CI, 0.43,9.91) point. Difference between groups was statistically significant (p=.032) |
| Blackstock, et.al.,  2013  Low | Blended learning  Simulation learning environment (SLEs) | **Practical exam** No significant differences in student competency between the SLE and control groups in either RCT.  RCT 1: The mean (SE) APP score for the 2 clinical examinations was 2.56 (0.05) for the SLE group and 2.61 (0.05) for the control group. The 95% CI of the difference in means was 0.09 to 0.17. Analysis of the APP scores for each of the 7 standards revealed no significant between group differences (all P 0.05)  RCT 2: The mean (SE) overall APP score was 3.02 (0.05) for the SLE and 2.80 (0.05) for the control group, 95% CI of the difference in means is 0.36 to 0.09, upper bound of the 0.4. Nevertheless, in 5 of 7 standards (not intervention or risk management), the SLE mean was significantly higher than that of the control (all P 0.05)  **Evaluation** Simulated learning environment; students in both RCTs showed significant change in all measures from baseline (all p > .01). Students rated SLE experience positively. In RCT 1, there was stability in self-ratings after the clinical immersion period. In RCT 2, students’ self-confidence declined marginally at the conclusion of the clinical immersion but remained comparable with the control group. Similarly, students in the control groups showed significant increasing confidence in all measures from start to end of the placement (allPG0.01). Students’ ratings of usefulness of the SLE immediately after the experience were as follows: RCT 1, 7.8 and RCT 2, 7.5. On completion of the clinical placement, ratings were less, and differences were significant (RCT 1, 6.4; Z=6.15; PG 0.01) (RCT 2,6.9; Z=3.63; PG 0.01). |
| Cantarero-Villanueva, et.al.,  2012  Low | Blended learning  Interactive website/app | **OSCE** Scores were significantly higher in the e-learning group than in the control group for skills in palpation ability and ultrasound assessment of the lumbopelvic area (both *p* < .001)  **Evaluation** No differences between the various aspects of the learning strategies. Both groups interested in participating in the study of each region using palpation and USI. Complexity of the learning goal: moderate, with no significant differences between groups. E-learning group reported a high level of satisfaction with the characteristics of the website. |
| da Costa  Vieira, et.al., 2017  Unclear | Blended learning  Video lectures | **Knowledge test** Significant acquisition of knowledge. Evaluating the correct answers; limited score in the pre-test (average grade 44.6%), significant (*p* < .001) improvement in post-test evaluation (average grade 73.9%).The correct pre-test (*p* = .556) and post-test (*p* = .729) evaluation and the retention of information (*p* = .408): not different between the two groups.  **Evaluation** Level of information retention was statistically similar between the traditional classroom group and e-learning group, encourages the use of e-learning in oncology. |
|  |  |  |

| **Author, year**  **Risk of bias** | **Digital learning design  Intervention** | **Results** |
| --- | --- | --- |
| Covill and Cook,  2019  High | Blended learning Flipped classroom | **MCQ** Correlation analysis of examination questions revealed high correlation with similar performance in all classes: Class A and B, r=0.62; Classes A and C, r=0.57; Class B and C, r=0.74.  **Evaluation** Students in both Class B and C: flipped experience met the learning objectives. Both cohorts: three favourite methods of pre-class activity; PowerPoint slides with voice and notes embedded, pre-recorded lectures and quizzes. Favourite in-class activities: Quiz review, response ware and large group discussion and lab/lecture. Not a preferred style in both groups: small group discussions and faculty answering questions related to pre-class activities. |
| Day, 2018 High | Blended learning Flipped classroom | **MCQ** Students in the flipped anatomy classroom: increase in semester average grades and performance on higher-level analytical questions. Long-term retention and knowledge transfer: analysed in a subsequent semester’s sequenced kinesiology course. Students from flipped anatomy classroom performed at a higher level in kinesiology. Lower performing students in a flipped anatomy class, outperformed their traditional anatomy class counterparts in anatomy semester grades accuracy on higher-level analytical anatomy multiple-choice questions and performance in subsequent course of kinesiology. |
| Deprey, 2018  High | Blended learning Flipped classroom | **MCQ** and short response questions: fFLIP (fully flip): the greatest improvements from examination 1 to examination 2; p ≤ .001, effect size(d) -1.37. The pFLIP (partly flip) cohort demonstrated significantlyy lower scores compared to full FLIP cohort on examination 2: p = .008, effect size (d) .76 |
| Fernandez-Lao, et.al., 2016 Low | Blended learning  Interactive website/app | **MCQ** No significant intergroup differences in the acquisition of theoretical knowledge (*p* = .089)  **OSCE** Scores were significantly higher in the experimental group than in the control group for the majority of items in the ultrasound assessment; positioning of patient (*p* < .001), positioning of ultrasound (*p* = .007), handling of ultrasound probe (*p* = .013) and global OSCE (*p* < .001), skills in palpation of the shoulder; position of patient (*p* = .009), direction of palpation contact (*p* = .021) and global OSCE (*p* = .034). There were no significant differences in the time required to perform the examination between groups in ultrasound (*p* = .944) and palpation (*p* = .393). No significant differences in the time required to perform the examination between groups in ultrasound and palpation.  **Evaluation** Results from the post-program survey assessing global satisfaction with the mobile application were high (8.200 ± .767) on an 11-point rating scale |
| Green & Whitburn, 2016 High | Blended learning  Online videos | **Practical and written exam** Statistically significant differences in scores comparing online videos and traditional classroom teaching; mean mark for practical test showed $F_{(2,459)}$=37.26, p<0.00 and final examination showed ${F(}_{2,455)}$ =10.86, p<0.00 between the cohorts.  **Evaluation** Some negative comments and some lower levels of intellectual stimulating. Fully blended version in 2015 resulted in more balanced comments about online content, but higher perceived workload (*p* ≤ .05). No difference in engagement in online discussions.  All cohorts listed practical classes as a major factor contributing to learning. |
| Huhn, et.al., 2013  High | Blended learning  Virtual patient simulation (VP) | **MCQ and OSCE** **Clinical reasoning** No significant differences in the Health Science Reasoning Test (HSRT) scores for method of instruction (f = .766, df=1, *p* = .386). There was a within-subjects effect for only the VP group (t=2.88, df=25, *p* = .008). A secondary analysis of the individual subscale scores of the HSRT completed to determine if improvements occurred in all of the subscales or specific subscales of the test There were no significant differences between groups for any of the subscales. However, for the VP group only, there was a within-sub-jects effect for the deduction (f=9.25, df=1, *p* = .004) and evaluation (f=7.21, df=1, *p* = .01) subscales  **Knowledge acquisition** There were no significant differences between the scores for the LGD (M = 74.07, SD = 8.47) and the VP group conditions (M = 77.65, SD = 7.95) (t = 1.58, df = 51, *p* = .059). A subset of test questions directly related to the cases completed also failed to identify between-group differences (t = 1.21, df = 51, *p* = .114)  **Transfer of Knowledge** No significant difference between the LGD (M = 88.79, SD = 24.23) and the VP group conditions (M = 89.67, SD = 8.91) (t = .792, df = 51, *p* = .214.). Outcomes for the VP group, less variable (9.9% variability) than the LGD (27% variability) group |

| **Author, year**  **Risk of bias** | **Digital learning design  Intervention** | **Results** |
| --- | --- | --- |
| Hyland, et.al., 2010  Unclear | Distance learning  Computer-assisted instruction (CAI) – learning management system | **MCQ** No significant difference was found between the groups for baseline knowledge, final exam scores or final course grades 52.0 ± 9.5 vs. 51.5 ± 12.7; *p* = .905), final exam scores (80.6 ± 7.8 vs. 85.1 ± 6.1; *p* = .073) or final course grades (90.2 ± 3.0 vs. 90.5 ± 3.1; *p* = .763) |
| Lazano-Lazano, et.al., 2020  Low | Blended learning design  Interactive website/app | **MCQ** Significant difference in the theoretical exam score (p<.001) in the favour of the Ecofisio (interactive website/app) group; mean 7.3 (SD 1.5).  **OSCE** significant difference in all components assessed during the OSCE stations (p<0.001 for all)  **Evaluation** Students satisfaction regarding the item: I believe the training is applicable (max 5 points): Intervention group: 3.3(0.8) and Control group: 3.0 (0.9). A total of 80% of the students gave scores of 8 or more on 0-10 points rating scale about their overall satisfaction level with the app |
| Maloney, et.al., 2013 (pilot)  Unclear | Blended learning  Pre-recorded video tutorial (PVT)  Student self-produced video (SSV) | **OSCE** No significant differences in clinical performance between the three practical teaching methods: traditional teaching (TRAD), pre-recorded video tutorial (PVT), student-produced self-video (SSV). Combining data for both skills A and B: p = .12.  **Evaluation** No difference for student ratings of satisfaction. Students’ ratings in perceived educational value with the teaching approach of pre-recorded video tutorial and student self-produced video being higher than “traditional” live tutoring; PVT, p = .003 and SSV, p = .007 groups comparison to TRAD, comparisons between PVT and SSV groups, equivalent, p = .80 |
| Maloney, et.al., 2013 (main)  Low | Blended learning  Student self-produced video (SSV) | **OSCE** Students received significantly higher scores in the OSCE when the examined clinical skill had been supplemented with a self- produced video of performance task (*p* = .048)  **Evaluation** Self-produced video of performance task utilized contributed to improvement in their clinical performance and their confidence for future clinical practice |
| Moore and Smith, 2012  Unclear | Blended learning  Video clips (video podcasting) | **MCQ** No significant difference in written and practical exam scores between instructional strategies for this cohort of DPT students; video podcasting (mean 7.22/20, SD 3.34) and traditional classroom teaching (Mean 5.69/20, SD 3.09), p≤.05. A significant difference in study time was observed only when comparing the podcast and live demonstration group based on group study time during transfer instruction (p= .004). The group receiving podcast demonstration reported more group study time (33.67 min, SD = 24.83) than the group receiving live demonstration of transfer skills (8.67 min, SD = 19.13).  **Evaluation** Respond to the statement ‘I would use this method again if available’; 94% in the podcast group agreed or strongly agreed, 87% in the live demonstration group either agreed or strongly agreed. The remaining 6% in the podcast group disagreed, while the remaining 13% in the live demonstration group were neutral. Total study time and study-alone time was similar between the podcast and live demonstration groups. A significant difference in study time was seen only when comparing the podcast and live demonstration groups based on group study time during transfers instruction (*p* = .004) examining group study time in the transfer training group. |
| Murray, et.al., 2014  High | Blended learning  Flipped classroom | **MCQ** No significant differences between teaching methods in any category of student outcomes, *p* = .19, M = 79.92 and SD = 5.91 (traditional classroom) and M = 83.6 and SD = 6.42 (flipped classroom) |
| Nicklen, et.al., 2016  Unclear | Blended learning  Remote online case-based learning | **MCQ** CBL median scores for the postintervention multiple‑choice test was comparable (Wilcoxon rank sum *p* = .61) (median/10 [range] intervention group: 9 [8–10] control group: 10 [7–10]). Of the 15 examinable learning objectives, 8 were significantly in favour of the control group, suggesting a greater perceived depth of learning.  **Evaluation** Eighty‑four percent of students (16/19) disagreed with the statement, ‘I enjoyed the method of CBL delivery’. Key themes identified from the focus group included: risks associated with the implementation of, challenges of communicating in, and flexibility offered by web‑based programs |
| **Author, year**  **Risk of bias** | **Digital learning design**  **Intervention** | **Results** |
| Noguera, et.al., 2013  High | Blended learning  3D interactive mobile application (app) | **MCQ** Significant relevance in learning outcomes using this tool. Knee and ankle; mean value and standard deviation of the first post-test scores: 6.87±0.8 for the experimental group and 4.8±0.8 for the control group to pre-test result, a significant difference was found between the two groups. Mean difference between groups: 2.07 (95% CI: 1.66–2.47) in a range of 0–8. A value of 100% corresponds to the eight questions answered correctly; significant increase over 25% of learning achievement for the experimental group.  Practical lesson about the knee and ankle: the proposed 3D m-learning tool was more effective than classical teaching methodology. Rating from the first post-test: no differences between genders (*p* = .98). First post-test: students in the experimental group obtained high percentages of correct answers, above 77%. Regarding the second practical lesson (about the pelvic zone), the mean and SD of the second post-test scores: 6.08±1.4 for the experimental group and 5.37±1.2 for the control group, significant difference between the two groups. The mean difference between the groups: 0.7 (95% CI: 0.3–1.37) in a range of 0–8: a small increase of about 8% of learning achievement for the experimental group (considering that 100% corresponds to all questions answered correctly). Practical lesson about the pelvic zone: 3D m-learning tool, better learning outcome compared to traditional methods, but less effect compared to the first practical lesson. No difference by gender (*p* = .48) in knowledge of pelvic zone anatomy (second post-test). Second post-test, students in experimental group: higher percentage of correct answers in questions 2, 3, 4, 5 and 8, lower percentages in questions 1 and 7. The same percentage as the control group in question 6.  **Evaluation** The evaluation was uniformly very positive; in general, the results did not show considerable deviations between groups (students and professors), *p* < .05. |
| Rocha, et.al., 2017  High | Blended learning  Educational video game (EVG) | **MCQ** Significant differences between experimental group (EG) and control group (CG) in specific knowledge test (*p* = .006); experimental group (EG); mean 9.07, SD ± 0.60) and control group (CG); mean 8.58, SD ± 0.64  **Evaluation** Results seem to indicate that the use of the education video game (EVG) can increase satisfaction with the discipline and perception of the learning content. |
| Silva, et.al., 2012  Unclear | Blended learning  Multimedia online tool | **Knowledge test** Online group performed significantly better than the control group (respectively, 7.75 (SD = 1.28) vs. 5.93 (SD = .72); *p* > .05). The use of a multimedia online resource had a positive impact on students’ learning in the respiratory therapy field. |
| Ulrich, et.al., 2019  Low | Blended learning  E-learning/Virtual reality, interactive learning environment 360° video | **MCQ** No statistically significant differences, f (2, 81) = 1.512, *p* = .227. The post-hoc test was also unable to identify any significant differences between 360° video and regular video (*p* = .135). Same non-significant results were found between 360° video and traditional teaching (*p* = .141) and between regular video and traditional teaching (*p* = .970). All treatment groups were equally effective. A significant difference found in communication, χ^2^(2) = 38.780, *p* < .000. The composite for emotions: significant difference between the three groups, χ^2^(2) = 18.221, *p* < .001. In the students’ perception of communication in the learning climate, 360° video (mean rank = 16.50) was outperformed by traditional teaching (mean rank = 39.35), U = 56, z = -5.36, *p* < .001. Likewise, regular video (mean rank = 16.00) was outperformed by traditional teaching (mean rank = 38.42), U= 54, z = -5.34, *p* < .001. No significant differences were found between 360° video (mean rank = 28.36) and regular video (mean rank = 27.63), U = 368, z = 78, *p* = .859. Regarding the students’ emotions about the learning environment, there was no significant difference between the 360° video (mean rank = 25.96) and traditional teaching (mean rank = 29.15), U = 321, z = 53, *p* = .452. However, the 360° video (mean rank = 35.43) outperformed the regular video (mean rank =20.30), U = 170, z = -3.507, *p* < .001. Likewise, the regular video (mean rank = 19.15) was outperformed by traditional teaching (mean rank = 35.15), U = 139, z = -3.782, *p* < .001. Even though the 360° video outperformed the regular video on emotions, it was not significantly more effective than traditional teaching.  **Evaluation** 360° video outperformed regular video only on the students’ emotions about the learning climate. 360° video and regular video were less effective than traditional teaching in students’ learning satisfaction. Comparing: traditional teaching is, on most constructs, equally or more effective. |
